# Management of epilepsy through indigenous traditional and Western approaches in Africa: A systematic review

**DOI:** 10.4102/hsag.v27i0.1984

**Published:** 2022-11-22

**Authors:** Qolile Chabangu, Maria S. Maputle, Rachel T. Lebese

**Affiliations:** 1 Department of Advanced Nursing, Faculty of Health Sciences, University of Venda, Thohoyandou, South Africa; 2 Research Office, Faculty of Health Sciences, University of Venda, Thohoyandou, South Africa

**Keywords:** epilepsy, indigenous traditional management, spiritual healers, traditional healers, effectiveness

## Abstract

**Background:**

Reaction to epilepsy management has been described as moulded by traditional beliefs, despite the reported progress of anti-epilepsy medication. In Africa, traditional healers are seen as essential in providing epilepsy care, yet little is known about their epilepsy care.

**Aim:**

This manuscript aimed to systematically review and summarise the various indigenous traditional and Western methods of epilepsy management and their effectiveness in Africa.

**Setting:**

This study is conducted in Africa.

**Methods:**

A systematic review was performed, searching MEDLINE (through PubMed), Google Scholar and ScienceDirect data from 2000 to December 2021. The search strategies used terms and medical subject headings ‘traditional methods’ AND ‘epilepsy’ AND ‘management’ AND ‘Africa’. The bibliography of the included articles was manually searched. Critical Appraisal Skills Programme and systematic reviews of randomised controlled trials tool were used to identify the validity of studies.

**Results:**

The search generated 17 927 articles. After screening for titles and abstracts, duplicate entries were removed and full texts of 22 articles were reviewed. After reading full texts, 12 articles met the inclusion criteria. The themes identified from synthesised data were indigenous traditional and Western methods of epilepsy management.

**Conclusion:**

Traditional and faith-based healers were perceived to provide frontline care for people living with epilepsy resulting in considerable delays in seeking anti-epilepsy medication initiation. Furthermore, taking anti-epilepsy treatment was not adequately adhered to.

**Contribution:**

Findings would contribute to the body of essential information to create awareness and upskill the community that epilepsy is like any medical condition that needs medical care.

## Introduction

Globally, according to the World Health Organization (WHO 2018), epilepsy is diagnosed in 2.4 million people yearly. Nearly 80% of the 50 million people currently living with epilepsy are in low- and middle-income countries. From a medical perspective, epilepsy is defined as an illness of the brain which is characterised by an event of unpredictable disturbance of the normal function called epileptic seizures (WHO 2012). The most readily identifiable seizures were divided into tonic, myoclonic, clonic, tonic-clonic, atonic, automatisms, hyperkinetic and epileptic spasms (Magazi, Nkohla & Mmako 2018). However, one should have one or more unprovoked seizures to be diagnosed as epileptic.

For many Africans, the response to epilepsy was made by indigenous beliefs amazingly related to each other one way or the other (Bhalla et al. 2014). Sub-Saharan Africa is a diverse continent that symbolises people from different cultural backgrounds. Therefore, there is a prevalent belief that epilepsy is a supernatural cause and not correctable with biomedical methods (Bhalla et al. 2014). The author looked at countries such as the Republic of Congo, Zimbabwe, Zambia, Namibia, Gambia, Uganda, Kenya, Tanzania and South Africa, whereby epilepsy was believed to be caused by witchcraft, an evil spirit, disobeying ancestors, punishment from God and African magic (Osakwe, Otte & Alo 2014). It was also thought that epilepsy was a contagious disease that can be transmitted through saliva, urine, blood and faeces.

In India and Nepal, epilepsy was also shaped by indigenous practices. During management, they would consult priests, some wore amulets to ward off evil spirits and some organised special prayers in the hope of a cure, casting anti-spell water for patients to drink and herbal medicine (Khwaya, Signh & Chaudhry 2007). However, in some African countries, anti-epilepsy medications were commonly used, but they also used prayers and herbal medicines to manage epilepsy (WHO 2012). Furthermore, in South Africa, public health facilities and private hospitals have made efforts to assist people with epilepsy. Despite the ‘bring epilepsy out of the shadow’ campaign, studies continue to show low uptake of anti-epilepsy drugs (Dewa 2012; Mutanana 2018). The Epilepsy Support Foundation Zimbabwe (2016) reported that about 86% of people living with epilepsy are not on anti-epilepsy medicine. Africans prefer to use the traditional and spiritual methods to manage epilepsy as they were groomed under certain beliefs (Dewa 2012). It was further documented that many people firmly believe that epilepsy was linked to a spiritual cause and about 70.5% of people consult traditional healers and their pastors first (Mohammed & Babikir 2013). It was the standard practice for people with epilepsy to consult biomedical practitioners for assistance at a later stage of the disease, which could contribute to complications (Bhalla et al. 2014).

## Methods

### Review question

The researcher conducted a systematic review by formulating a review question, conducting a search strategy, critical appraisal, data extraction and synthesis. The formulated review question was as follows: ‘What is the perceived effectiveness of indigenous management of epilepsy by rural communities?’ This was carried out by screening the titles of studies in the search engine, followed by screening the abstract and subsequently the selected complete text (PRISMA). The reviewer extracted the necessary data into a form designed in the protocol to summarise the included studies and assess bias. Authors further identified the quality of the available evidence and developed tables and text that synthesised the evidence (CASP [Critical Appraisal Skills Programme]).

### Search strategy

The literature search was performed in MEDLINE (through PubMed), Google Scholar (through search engine and library databases articles) and ScienceDirect for data from December 2000 to December 2021. The following terms and medical subject heading were used in the search strategies: ‘traditional methods’ AND ‘western methods’ AND ‘epilepsy’ AND ‘management’ AND ‘Africa’. The librarian assisted with the application of different search strategies for different databases used in this review. The strategies for other databases were available on request. The bibliography of the included articles was manually searched. Two authors independently evaluated the titles and abstracts of all identified studies in the search based on the above-mentioned terms and Medical Subject Headings (MeSH).

### Inclusion and exclusion

The authors included studies documenting the various traditional and Western methods of epilepsy management and its effectiveness in Africa. All published and full text articles from December 2000 to December 2021 focus on the traditional methods and Western methods of epilepsy management in Africa. Studies targeted and included adults of age 18 and above with new or chronic epilepsy, family members, caregivers of those with epilepsy and indigenous traditional healthcare professionals. All studies were written in English and were provided in full publications of peer-reviewed journals. Studies older than 20 years, abstract, incomplete, duplicate and unpublished articles were excluded.

### Study selection

**FIGURE 1:**
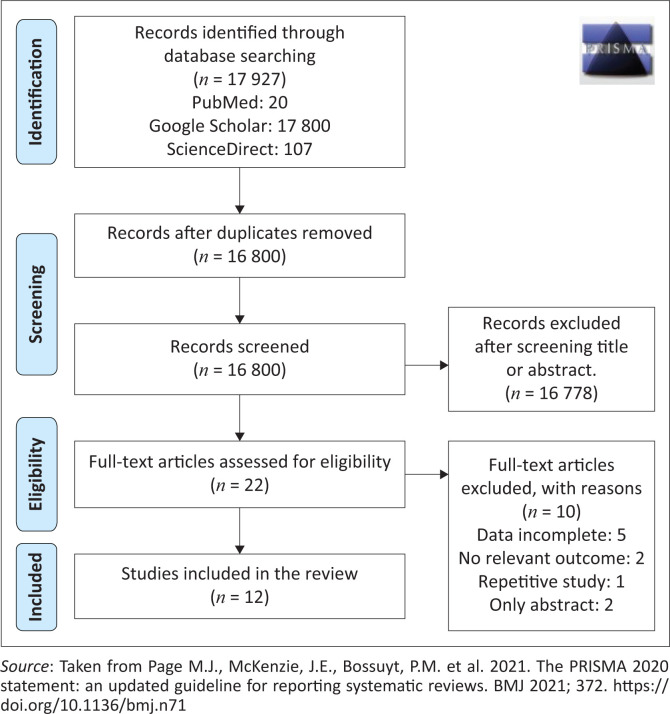
PRISMA diagram.

The authors reviewed African studies from December 2000 to December 2021 by reading the titles and abstracts before reading through the article. Duplicates and the studies that consisted of abstracts only were removed. The general search studies were 17 927 (PubMed = 20, Google Scholar = 17 800 and ScienceDirect = 107). The records after removal of duplicates remained 16 800 articles; after title and abstract screening, 16 778 articles were excluded and the authors remained with 22 articles to be assessed for eligibility. After assessing eligibility, 10 articles were excluded because of incomplete data, no relevant outcome, repetitive study and only abstract; however, only 12 studies met the criteria and were included for the study (Figure 1).

### Study appraisal

This study’s appraisal was carried out through CASP, a checklist that allows the author to check the study’s methodological quality against fixed criteria. The author used the CASP appraisal programme checklist to identify and appraise the included studies (represented in Table 1). The CASP checklist consisted of 10 questions. For each study, the following questions were asked:

1. Was there a clear statement of the aims of the study?
2. Is a qualitative or quantitative methodology appropriate? The introduction, title, abstract and background should indicate whether the proper qualitative or quantitative approach was appropriate.
3. Were the research designs appropriate to address the aims of the research?
4. Was the recruitment strategy appropriate to the aims of the research?
5. Was the data collected in a way that addressed the research issue?
6. Has the relationship between researcher and participants been adequately considered?
7. Have ethical issues been taken into consideration?
8. Was the data analysis sufficiently rigorous? The methods section provides sufficient information about how data were analysed.
9. Is there a clear statement of findings? Looking at the results section, have the authors provided their interpretation of the data.
10. How valuable is the research? Check if the studies add value to the research question.

**TABLE 1:** Critical appraisal skills programme study appraisal.

Author	Title	Designs	%
Anand et al.	Epilepsy and traditional healers in the Republic of Guinea	Qualitative	90
Keikelema et al.	‘The others look at you as if you are a grave’ A qualitative study of subjective experiences of patients with epilepsy regarding their treatment and care in Cape Town	Qualitative	78
Dolo et al.	Community perceptions of epilepsy and its treatment in an Onchocerciass endemic region in the Ituri Republic of Congo	Qualitative	85
Mutanana	Indigenous practices for suitable management of epilepsy in Zimbabwe	Mixed method study	92
Mutanana et al.	Challenges associated with anti-epilepsy medication and use of complementary or alternative medicine among people with epilepsy in rural communities of Zimbabwe	Qualitative	88
Winker et al.	Attitude towards African traditional medicine and Christian spiritual healing regarding the treatment of epilepsy in a rural community of northern Tanzania	Qualitative	90
Kendall-Taylor et al.	Comparing characteristics of epilepsy treatment provider on the coast: Implication for treatment-seeking and intervention.	Qualitative	87
Mohammed and Babikir	Traditional and spiritual medicine among Sudanese children with epilepsy	Quantitative	88
Rutebemberwa et al.	Biomedical drugs and traditional treatment in care-seeking pathways for adults with epilepsy in Masindi District, western Uganda, a household survey	Qualitative	92
Coleman et al.	The treatment gap and primary healthcare for people with epilepsy in rural Gambia	Mixed method	96
Du Toit and Pretorius	Seizure in Namibia: A study of the traditional health practitioner	Qualitative	86
Baskind and Birbeck	Epilepsy care in Zambia: A study of a traditional healer	Qualitative	78

The authors used the percentage to rate the study validity as the CASP has 10 questions, each considered 10% if the study correctly met the criteria. The rating was performed manually by the authors. When the percentages are 80% and above, the study validity is higher, and when it’s 70% to 50%, it is considered average; from 49% downward, the study is not valuable enough to be included. Through the critical appraisal this systematic review adhered to rigour, as only studies included in this research adhered to the CASP checklist. The included studies were of high rigour as they scored the critical appraisal score of 78% to 96%.

### Data extraction and characteristics of the selected studies

The extracted data, from the studies by the authors, considered the characteristics; author(s), country and year, review purpose, design, sample, findings and limitations.

## Findings

This study aims to systematically review the various traditional and Western methods of epilepsy management and their effectiveness in Africa. The results were based on the findings of studies conducted in the Republic of Congo, Zimbabwe, Zambia, Namibia, Gambia, Uganda, Kenya, Tanzania and South Africa. The management and perceptions of epilepsy have been found to differ in African cultures (Millogo et al. 2019). Although the origin and causes of epilepsy remain unknown, an evil spirit is thought to cause epilepsy, still influencing people’s perception of epilepsy management (Siriba 2014). However, the management of epilepsy in public health sectors is affected by several factors such as a lack of trained staff in epilepsy, beliefs about epilepsy, unavailability of drugs and poverty (Eastman 2005; Williams, Nefdt & Wilmshurst 2015). In addition, a study in South Africa by Keikelema and Swartz (2016) shows that some people with epilepsy combine Western and traditional methods to manage epilepsy. The following themes were identified from the synthesised data: traditional methods of epilepsy management and Western methods of epilepsy management.

### Theme 1: Traditional methods of epilepsy management

In this study, a traditional health practitioner is recognised in the community. They provide healthcare through animals, plants and other methods that are based on cultural, religious and social elements. Traditional methods are diverse, including prayers, herbalists, spiritualists and diviners (Newton & Garcia 2012). Traditional health practitioners are essential in managing epilepsy in many low- and middle-income countries. However, in Africa, traditional healers are one of the primary sources of healthcare (Newton & Garcia 2012).

**TABLE 2:** Characteristics of the included studies.

Author, Country Year	Purpose	Design	Sample	Findings	Limitation
Anand et al., Guinea March 2019	To characterise the reason, extent and impact of traditional medicine use among people with epilepsy in the Republic of Guinea	A qualitative design	A total of 132 people living with epilepsy and caregivers.	Most people in Guinea consult traditional healers as their frontline care for people with epilepsy and seek medical care later in the failure of traditional attempts.	No limitation was indicated in the study.
Keikelema and Swartz, South Africa 2016	To explore patients’ experiences regarding their treatment and care in Cape Town	Qualitative design	A total of 12 people living with epilepsy were interviewed. Snowball and purposive sampling were used.	Anti-epilepsy drugs are commonly used; however, the behaviour of the healthcare practitioner results in patients losing interest in taking medication.	The study sample was from a small area and cannot be generalised.
Dolo et al., Congo 2018	To investigate the perceptions and experiences regarding epilepsy and its treatment among community leaders, their families, traditional healers and health professionals	Qualitative design	A total of 14 focus group discussions involving 60 community leaders. 35 PLWE and their family, 6 traditional healers, and 39 semi-structured interviews with PLWE, family members and health professionals.	Epilepsy is treated using AEM and traditional treatment using sprays (in the churches), plants (mainly one plant called ‘*dodo*’), incantations (to chase away evil spirits) and bathing in the river.	No limitation
Mutanana, Zimbabwe 2017	To analyse challenges associated with anti-epilepsy medication and complementary medicines among people with epilepsy in rural communities in Zimbabwe	Qualitative design	Target populations are people living with epilepsy and their caregivers. Snowball sampling was used to sample 15 people living with epilepsy and five caregivers. Purposive sampling was used to sample two traditional healers, two faith-based healers and two psychiatric nurses.	They consult traditional and faith healers because they are accessible. Participants agree that traditional and spiritual medicines are effective in epilepsy management. They were satisfied with the service received by traditional and spiritual healers in the communities of Zimbabwe.	The study suffered from methodological limitations in the sample size. It only focuses on one community.
Mutanana, Zimbabwe 2018	The study aimed to explore indigenous practices for management of epilepsy in Zimbabwe	Mixed methods	A sample of 150 family members 170 people living with epilepsy were identified using proportional stratified random sampling. Doctors, nurses and psychologists were identified using purposive sampling, and traditional healers, herbalists; Christian healers were identified using snowball sampling.	The findings revealed that people with epilepsy are not on anti-epilepsy treatment and they firmly believe that epilepsy is caused by witchcraft and an evil spirit. They think bio-medication does not help treat the disease; however, people with epilepsy have resorted to indigenous practices of epilepsy management. Indigenous practices are very effective in epilepsy management. And bio-medication is not helpful.	The study focused on people living with epilepsy who are on treatment and residing in Zimbabwe getting counselling and help from the Epilepsy Support Foundation of Zimbabwe.
Winker et al., Tanzania 2009	To explore the prevailing attitude towards traditional medicine for the treatment of epilepsy in a rural area of Northern Tanzania	Qualitative	A total of 167 people were interviewed 59 people living with epilepsy, 62 relatives and 46 villagers.	Most people were convinced that traditional healing methods may successfully cure epilepsy, while some thought prayer would cure or treat its causes and symptoms.	No limitation was indicated in the study.
Kendall-Taylor et al., Kenya 2009	To examine how treatment seeking may be facilitated or deterred by the characteristic of available treatment options	Qualitative	Eight traditional healers and 12 biomedical health workers were interviewed using convenience and snowball sampling.	Traditional healers were found to be having more influence in the treatment of epilepsy and their medicine differs from the one in the facilities by causation, social role, referral practices and system of payment.	A lack of data from the individual decision-maker.
Coleman et al., Gambia 2002	To study primary level management for people with epilepsy in the rural Gambia using a community survey	Mixed method	Sixty-nine people were surveyed.	In Gambia, treatment for epilepsy is costly, according to the study. Therefore, the patient goes for what they can afford during a seizure.	No limitation was indicated in the study.
DuToit and Pretorius, Namibia 2018	To understand traditional health practitioners, perception and experience in the delivery seizure in Namibia	Qualitative	Eleven traditional health practitioners were interviewed. Thematic was used.	Treatment is based on the causes of the illness rituals, and herbal is prepared through the guidance of the spirit, making the treatment unique to everyone.	With a small study sample size, the purpose was to generate depth rather than breadth. The study focused on developing information-rich findings on the perceptions and experiences of THPs in managing seizures.
Baskind and Birbeck, Zambia 2005	To better understand the epilepsy care delivered by traditional healers in Zambia	Qualitative	Focus group discussion with 10 traditional healers. Four physicians and eight traditional healers were interviewed one on one.	Epilepsy is treated with an antidote comprising the same ingredients used to bewitch the patient, failure to identify the causes lead to treatment failure.	The qualitative method does not seek to find a representative sample of informants.
Rutebemberwa et al., Uganda 2020	To identify the healthcare provider where patients with epilepsy sought care and what treatment they received	Qualitative	A total of 305 households with people living with epilepsy were surveyed using a questionnaire.	The patient seeks treatment from multiple providers, with the public sector attending to the most considerable proportion of patients.	No limitations
Mohammed and Babikir, Sudan 2013	To study the impact of spirituality on the explanation of epilepsy aetiology and traditional medicine used in the management of epilepsy in Sudan	Quantitative	A total of 180 caregivers, which include 165 mothers, 10 fathers, 2 grandmothers, and 3 relatives.	The population in Sudan uses traditional and spiritual medicine to meet their primary healthcare needs because it is accessible, affordable and part of their belief system.	No limitations

AEM, Anti-epileptic Medicine; PLWE, people living with epilepsy; THP, Traditional Health Practitioner.

#### Sub-theme 1.1: Christian healers

The findings of this study reveal that in Africa, there are many Christian healers. They manage epilepsy by praying as prayer warriors at church and trying to do away with the demons or evil spirits (Mutanana 2018). However, prayers are made unique in African countries, and they are on an emerging indigenous understanding of Christians, which is based on the emergent African Pentecostal movement (Mutanana 2018). Furthermore, Christian healers believe their healing power comes from God and sometimes combines ancestral and Christian Holy Spirit (Karim, Ziqubu-Page & Arendse 1994). Services offered by Christian healers are diagnostic and curative (Truter 2007). The Christian healers show that they can heal epilepsy and have assisted many people through prayers, prophecies and prayed water (Dolo et al. 2018).

The findings in West and East Africa show that prayers, magic, charms, taboos and religious ceremonies to take away a spirit are effective. Still, one has to believe in their God (Allah) and follow the procedure and guidelines from the Quran (Abdoulie et al. 2020). However, the standard spiritual techniques used to manage epilepsy by the Sudanese are incantations, spitting curses and ritual incensing. About 42.5% show that they started consulting Christian healers before seeking medical attention (Abdoulie et al. 2020; Mohammed & Babikir 2013). Furthermore, results show that people living with epilepsy in Zimbabwe seek divine intervention at the churches led by prophets (Mutanana 2018). Some people with epilepsy strongly believe their prophets are healing them, while others suggest that these are hallucinations. As this disease has been associated with the evil spirit, they have no options but to use traditional methods to manage the condition (Keikelema 2015; Mutanana 2018).

#### Sub-theme 1.2: Traditional healers

The findings of this study show that traditional indigenous practices shape epilepsy; these provide frontline care for people living with epilepsy (Mutanana 2017). However, results show that epilepsy can be cured only if the patient has never had fire burns before, while some participants show it is not curable (Baskind & Birbeck 2005; Mutanana 2017). Traditional healers are accessible in Africa with at least one traditional healer in each community compared with only four neurologists for each country (Anand et al. 2019). As a result, people seek treatment at health facilities as its easily accessible; however, there is a shortage of medication in clinics and hospitals. Many people do not use anti-epilepsy drugs (Anand et al. 2019).

In Africa, the management of seizures is unique to each person and is always led by what the spirits tell about the person and the cause of the illness (Du Toit & Pretorius 2018). Each traditional health practitioner employs different ceremonies and herbal preparations during treatment as instructed by the spirit (Du Toit & Pretorius 2018). Other practices include using plant materials, insects, healing prayers and laying hands (Du Toit & Pretorius 2018). Another type of herbal medicine belongs to the supernatural realm and is only known to traditional healers. As epilepsy is caused by witchcraft, it is assumed that epilepsy requires some antidote against the ‘poison of witches’ (Du Toit & Pretorius 2018). Witchcraft-induced seizures can be cured by treatment with an antidote comprising of the same ingredients used in the original witchcraft (Baskind & Birbeck 2005). Treatment failures occur when the healer cannot identify and obtain the right ingredients (Baskind & Birbeck 2005). Only traditional healers can detect the poison and find the herbal antidote (Du Toit & Pretorius 2018; Winkler et al. 2010). These herbs grow in specific places, and it’s not affected by evil spirits. (Du Toit & Pretorius 2018; Winkler et al. 2010). Treatment with these plants is much more expensive compared with ordinary herbal medicine.

In Tanzania, the healer produces powder from roots, barks and leaves of trees and plants, which are unknown to the receiver but usually are available at the market for the management of epilepsy (Winkler et al. 2010). Some said it is added to porridge or tea, while others reported a topical application, sometimes into skin lacerations set just for this purpose (Winkler et al. 2010). The healer makes minor cuts into the skin of body parts (scarification) that are affected by seizures (Winkler et al. 2010). These cuts are sometimes used as a depot for herbal medicine; in other cases, the scarification itself represents the treatment. In most cases, the cuts are set on the head and face but may be found all over the body (Winkler et al. 2010).

Traditional healing in Guinea is classified into two categories: treatment administration during the seizure and prevention and cure of epilepsy (Anand et al. 2019). During seizures, traditional healers put garlic, orange, alcohol or a paste made of grains into the nose or mouth of the seizing person and read Koranic scripture to end seizures (Anand et al. 2019). As prevention for people with epilepsy, traditional healers use the application of *talisman* (French), *gris-gris* (Malinke) or sebe (Susu), rubbing Koranic scripture over the body and reciting Koranic scripture in water and giving it as a drink. (Anand et al. 2019; Coleman, Loppy & Walraven 2002). The treatment in the Gambia includes reading from the Koran, sometimes written down and sewn into cloth or leather amulets that had to be worn (Coleman et al. 2002).

The findings in Zimbabwe indicate that a herbalist uses herbs known as *Mupingangozi* (the herbs are found in Mozambique) to manage epilepsy. The herbs are mixed with a cup of boiled water given to people with epilepsy then the seizure will commence releasing the foam. Therefore, the foam has been shown to be a pigeon that will take the foam and fly away, which will end epilepsy (Mutanana 2018). In a study it was found that to manage epilepsy the herbalist wakes in the morning before anyone else and takes the grass on the road mixed with cold water then gives it to the patient biting the spoon, by the time the spoon is removed, epilepsy will be cured (Mutanana 2018). Furthermore, Zimbabweans reveal that many communities believe in traditional practices and consequently resort to traditional and spiritual medicines (Mutanana 2017).

Some studies reported availability and proved that a traditional religious healer helps to manage epilepsy (Keikelema & Swartz 2016). Many people in the community showed that they were satisfied with the services offered by traditional and religious healers (Keikelema & Swartz 2016). Nevertheless, the management of epilepsy by traditional healers has been reported effectively by the communities, and people have a positive attitude towards the African management because it has to do with their beliefs on epilepsy (Mutanana 2017). All the healers claimed that seizures are completely healed once they treat a person. Success is measured by the complete absence of episodes as reported by the person (Du Toit & Pretorius 2018). Most people consult traditional healers as their frontline care for patients with epilepsy and delay seeking medical care and anti-epilepsy drug initiation (Anand et al. 2019).

### Theme 2: Western methods of epilepsy management

**Anti-epilepsy medication:** There are more than 20 prescriptions of anti-epilepsy drugs available in Africa. One option depends on lifestyle, type of seizure, age and how often the person has a seizure (Federation of Disability Organisations in Malawi (FEDOMA) 2011). Drug treatment is also given according to the individual’s characteristics and the patient’s type of seizure (Tuan 2010).

Phenytoin and phenobarbital are the most used epilepsy drugs. Phenobarbital was considered as more secure in the country’s primary care system (Tuan 2010). In Africa, phenytoin and phenobarbital are the cheapest and prescribed in 65% to 85% of patients and considered most effective. However, other epileptic drugs, including carbamazepine and valproic acid are also accessible but are very expensive (WHO 2012). All medications are widely dispensed at the African pharmacy and healthcare facilities. The anti-epileptic drug has about a 60% to 70% chance of treating seizures (Keikelema & Swartz 2016). Patient Education Institution supports effective anti-epileptic medication and about 80% of patients with epilepsy can have seizures controlled with medication. Anti-epilepsy drugs are recommended as less harmful because they are scientifically proven, unlike indigenous practices (Anand et al. 2019). The African beliefs in treating epilepsy and the side effects of drugs result in the underutilisation of anti-epileptic medication. Firstly, many people consult African healers when there is no relief from an African cure, they seek healthcare from Western medicine (Dolo et al. 2018). A study shows that some people with epilepsy use anti-epilepsy medication and indigenous practices; however, some leave anti-epilepsy medication completely for indigenous practices (Mutanana 2018). In South Africa, efforts are being made to assist people living with epilepsy at government hospitals, clinics and non-governmental organisations such as the Zimbabwe Epilepsy Foundation. However, in spite of these efforts still there is less uptake of anti-epilepsy medication (Kendall-Taylor et al. 2009; Rutebemberwa et al. 2020). Traditional medicine can play a significant role in treating epilepsy when used together with modern approaches.

## Discussion

The study aims to systematically review the various traditional and Western methods of epilepsy management and their effectiveness in Africa. The following themes were identified from raw data: traditional methods of epilepsy management and Western methods of epilepsy management.

In Africa, two approaches are used, namely traditional and Western. The traditional approach includes traditional healers, herbalists, pastors and prophets. Traditional healers are embedded within communities and are instrumental in defining and disseminating beliefs and attitudes regarding illness (Puckree et al. 2002). Traditional healers are usually more geographically accessible than Western healthcare providers (Puckree et al. 2002), and they can communicate more readily with patients and families than their Western counterparts (Baskind & Birbeck 2005). Regarding epilepsy management, traditional healers use plants and animals to treat epilepsy while pastors and prophets use oil, holy water, prayers and sprays. These practices were used to take away the evil spirit.

In contrast, Western healthcare practitioners, including nurses and doctors, provide a Western approach. They prescribe anti-epilepsy medication, namely phenobarbital, phenytoin, carbamazepine, valproic acid and diazepam, to manage epilepsy. Anti-epilepsy medications are underutilised because of the cost and are not easily accessible in other communities. It is proven that anti-epilepsy medicines can control seizures. However, there is no proof of evidence on traditional medicine even though many people still consult traditional healers and Christian healers to manage epilepsy. There seems to be limited community health literacy about epilepsy medication and its side effect, as evidenced by defaulting treatment and consultation of traditional healers for management, especially after noticing the side effects of the medicine. People with epilepsy seek medical health assistance after traditional and Christian healers fail to manage their condition. Even when PLWE can access medical care, they may simultaneously or first seek care from traditional healers.

The review had limitations that include a large variation, which may be because of the search strategy that was broad. Also, not all African countries were represented in this review.

## Conclusion

People living with epilepsy strongly believe that epilepsy is a spiritual issue and treatment should be carried out through herbs and prayers. Before seeking Western medical care, many patients consulted traditional and spiritual healers for epilepsy management. They were satisfied with the services offered by traditional and spiritual healers. Anti-epilepsy treatment was not adequately adhered to in Africa because of people’s perceptions and beliefs about epilepsy.

## Recommendations

As most people from the rural communities were consulting the indigenous traditional healers, it is recommended that they must strengthen awareness to community members to consult the health professionals for anti-epilepsy. The medical and indigenous practitioners must work as a team to ensure sustainable management of epilepsy. The establishment of collaboration between indigenous traditional and health practitioners should be encouraged. To achieve effectiveness, research is needed to assess the impact of such collaborations between biomedical services and traditional healers on epilepsy treatment and care. The basic knowledge, attitude and skills of indigenous traditional practitioners must be upskilled by healthcare practitioners in epilepsy management. This would assist the community to avoid any harmful practices.
